# The influence of baseline risk on the relation between HbA1c and risk for new cardiovascular events and mortality in patients with type 2 diabetes and symptomatic cardiovascular disease

**DOI:** 10.1186/s12933-016-0418-1

**Published:** 2016-07-19

**Authors:** Sophie H. Bots, Yolanda van der Graaf, Hendrik M. W. Nathoe, Gert Jan de Borst, Jaap L. Kappelle, Frank L. J. Visseren, Jan Westerink, on behalf of the SMART Study Group

**Affiliations:** 1 University College Utrecht, Utrecht University, Utrecht, The Netherlands; 2 Julius Center for Health Sciences and Primary Care, University Medical Center Utrecht, Utrecht, The Netherlands; 3 Department of Cardiology, University Medical Center Utrecht, Utrecht, The Netherlands; 4 Department of Vascular Surgery, University Medical Center Utrecht, Utrecht, The Netherlands; 5 Department of Neurology, Brain Center Rudolf Magnus, University Medical Center Utrecht, Utrecht, The Netherlands; 6 Department of Vascular Medicine, University Medical Center Utrecht, PO Box 85500, 3508 GA Utrecht, The Netherlands

**Keywords:** HbA1c, Cardiovascular disease, High risk population, Type 2 diabetes, Glycaemic control

## Abstract

**Background:**

Strict glycaemic control in patients with type 2 diabetes has proven to have microvascular benefits while the effects on CVD and mortality are less clear, especially in high risk patients. Whether strict glycaemic control would reduce the risk of future CVD or mortality in patients with type 2 diabetes and pre-existing CVD, is unknown. This study aims to evaluate whether the relation between baseline HbA1c and new cardiovascular events or mortality in patients with type 2 diabetes and pre-existing cardiovascular disease (CVD) is modified by baseline vascular risk.

**Methods:**

A cohort of 1096 patients with type 2 diabetes and CVD from the Second Manifestations of ARTerial Disease (SMART) study was followed. The relation between HbA1c at baseline and future vascular events (composite of myocardial infarction, stroke and vascular mortality) and all-cause mortality was evaluated with Cox proportional hazard analyses in a population that was stratified for baseline risk for vascular events as calculated with the SMART risk score. The mean follow-up duration was 6.9 years for all-cause mortality and 6.4 years for vascular events, in which period 243 and 223 cases were reported, respectively.

**Results:**

A 1 % increase in HbA1c was associated with a higher risk for all-cause mortality (HR 1.18, 95 % CI 1.06–1.31). This association was also found in the highest SMART risk quartile (HR 1.33, 95 % CI 1.11–1.60). There was no relation between HbA1c and the occurrence of cardiovascular events during follow-up (HR 1.03, 95 % CI 0.91–1.16). The interaction term between HbA1c and SMART risk score was not significantly related to any of the outcomes.

**Conclusion:**

In patients with type 2 diabetes and CVD, HbA1c is related to the risk of all-cause mortality, but not to the risk of cardiovascular events. The relation between HbA1c and all-cause mortality in patients with type 2 diabetes and vascular disease is not dependent on baseline vascular risk.

## Background

Cardiovascular disease (CVD) is a major healthcare problem [1], especially in high income countries [2, 3], and it remains the most common cause of death and disability in patients with type 2 diabetes [4]. As the number of patients with type 2 diabetes is expected to grow to 592 million worldwide by 2035 [5], it is relevant to expand the understanding of the role of type 2 diabetes in the development and progression of CVD.

In cohort studies with patients with type 2 diabetes, poor glycaemic control, as measured by HbA1c, is associated with an increased risk for cardiovascular disease [6–8]. The relationship between increasing plasma HbA1c levels and a higher risk for incident macrovascular and microvascular disease is most prominent above a HbA1c of 7.0 % (53 mmol/mol) for macrovascular and 6.5 % (48 mmol/mol) for microvascular disease [9].

In clinical trials, lowering HbA1c levels in patients with type 2 diabetes has beneficial effects on incident microvascular complications [10–12], while the effect on macrovascular complications is less clear [10–13]. Follow-up analyses of these trials display positive effects of strict glycaemic control [14–16] as well as the absence of such benefits [14, 17] or even adverse effects [16]. Recent meta-analyses seem to support the more negative results and state that strict glucose regimes might not be the optimal treatment to reduce vascular risk for patients with type 2 diabetes [18, 19].

It has been suggested that these different findings might be explained by differences in patient characteristics between the studies [20]. It seems that healthier patients (younger, shorter history of CVD and/or type 2 diabetes, lower HbA1c at baseline) benefit more from strict glycaemic control than their older counterparts with a longer history of disease (CVD and/or type 2 diabetes) and a higher baseline HbA1c [13, 15–17].

These findings suggest that strict glycaemic control might not be beneficial in high risk patients, including patients with type 2 diabetes and cardiovascular disease [20]. However, little is known about the relation between HbA1c and risk for new cardiovascular events in these high risk patients [21]. As patients with type 2 diabetes and pre-existing CVD are at very high risk for new cardiovascular events and death, it is relevant to investigate whether glycaemic control remains an important amendable risk factor in these patients.

The recently published SMART (Second Manifestations of ARTerial Disease) risk score predicts 10-year risk of recurrent major vascular events and vascular mortality in patients with cardiovascular disease [22]. Their work showed that patients with a history of cardiovascular disease do not, as was previously assumed, always classify as high-risk patients (defined as having a 10-year risk of above 20 %), as the 10-year risk for recurrent events in their cohort ranged from 6 to 44 % [22]. The SMART risk score enables clinicians to quantify risk in individual patients and to identify those at the highest risk. We therefore investigated the relation between HbA1c and new cardiovascular events and mortality in patients with type 2 diabetes and CVD, stratified by their baseline risk for new cardiovascular events and mortality as calculated by the SMART risk score.

## Methods

### Study population

For this study we used data from patients with type 2 diabetes and cardiovascular disease enrolled in the Second Manifestations of ARTerial Disease (SMART) cohort. The design and rationale of the SMART study have been described previously [23]. To shortly summarise, patients aged 18–79 who are referred to the University Medical Center Utrecht with atherosclerotic vascular disease or for treatment of cardiovascular risk factors are included in the database. Physical and laboratory examinations are performed in the hospital after an at least 8-h fast. Information about a history of CVD, vascular risk factors and a detailed medical history are obtained via a questionnaire. Follow-up information is obtained via questionnaires sent to patients every 6 months or via the general practitioner (GP) if the patient is unwilling to fill out questionnaires and gives consent for GP consulting. The ethics committee of the University Medical Center Utrecht approved this study, all eligible patients received written and oral information about the study and all included participants gave informed consent [23].

For the current study, patients with type 2 diabetes and clinically manifest vascular disease at baseline were selected (n = 1205). Patients with a missing high-sensitivity C-reactive protein (hsCRP) value (n = 22) or a hsCRP value above 15 mg/L (n = 83) were excluded. The latter were excluded because we think such a high hsCRP value is more indicative of infection or a different cause of inflammation than the low-grade inflammation seen in patients with atherosclerosis [24]. Finally, we recalculated missing data regarding LDL using the Friedewald formula. Data that remained missing after this procedure (n = 6) was imputed. The participants for whom the SMART risk score could not be calculated (n = 4) were also excluded, bringing the final study population to 1096 participants.

### Study definitions

Diabetes mellitus was defined as a referral diagnosis of type 2 diabetes, self-reported type 2 diabetes, a fasting serum glucose ≥7.0 mmol/l at inclusion with initiation of glucose lowering treatment within 1 year or the use of oral anti-hyperglycemic agents or insulin at baseline. Participants with known type 1 diabetes were excluded from our analyses. History of clinically manifest vascular disease is defined as ever having had clinical manifest vascular disease [23]. HbA1c was studied as a continuous variable.

The cardiovascular outcomes defined are myocardial infarction (MI), ischemic stroke, the composite of major cardiovascular events, cardiovascular mortality, and all-cause mortality. Their definitions have been described elsewhere [23]. Each notification of a possible event is thoroughly checked by collecting all available information about the patient regarding the event. This includes all correspondence and documented investigations concerned with the particular event. All events were audited by three independent committee members of the Outcome Committee.

### Data analyses

Data are presented as mean ± standard deviation (SD) or median with interquartile range for variables with a skewed distribution. For all 1096 participants, the SMART risk score was calculated using the equation described by Dorresteijn et al. [22]. This equation predicts the 10-year risk of recurrent major vascular events (myocardial infarction, stroke and vascular death) in patients with CVD [22]. All analyses were performed for the whole cohort and in quartiles (Q1-Q4) of SMART risk. Non-HDL cholesterol was calculated for all patients by subtracting the measured HDL cholesterol from the measured total cholesterol value. To assess whether baseline risk as defined by the SMART risk score has a modifying effect on the relation between HbA1c and new outcomes, the interaction term between HbA1c and SMART risk score was used. We considered the effect modification by the SMART risk score significant when the p value of the interaction term was <0.05. Cox regression modelling was used to assess the relation between HbA1c and cardiovascular outcomes and mortality. Cubic spline analysis was rejected based on the inability of this technique to measure effect modification. Results are given as hazard ratios with 95 % confidence interval, and denote the increase in risk for a cardiovascular outcome or mortality related to a 1 % increase in HbA1c. Three models were constructed. The first model was a univariate model which only included HbA1c. The second model was adjusted for sex and age, and the third model was additionally adjusted for the known confounders current smoking, non-HDL cholesterol, diabetes duration, systolic blood pressure and eGFR (MDRD). We performed sensitivity analyses including the earlier excluded patients with a hsCRP value above 15 mg/L (n = 83) and additional analyses adjusted for all components of the SMART risk score. All analyses were performed in an imputed dataset, as missing data is seldom missing at random. We imputed missing data on eGRF(1, 0.09 %), LDL cholesterol (6, 0.5 %) and HbA1c (100, 9.1 %). All analyses were performed using SPSS 21 (IBM, New York). Graphs were created using R 3.1.1, (The R Foundation for Statistical Computing, Austria).

## Results

### Baseline

The mean age of the participants in the cohort was 62.6 ± 8.8 years and 76 % of the participants were male. The SMART risk score ranged from a predicted 5–100 % 10-year risk for recurrent vascular events. The creation of quartiles based on the SMART risk score resulted in four groups with a SMART risk score range of 5–16, 16–24, 24–37 and 37–100 % respectively (Table 1).

**Table 1:** Baseline characteristics of the whole cohort and stratified by vascular risk

	Whole cohort	Q1	Q2	Q3	Q4
	n = 1072	n = 268	n = 268	n = 269	n = 268
SMART risk score (% 10 year risk for recurrent vascular events)		5–16	16–24	24–37	37–98
Age (years)	62.6 ± 8.8	55.8 ± 7.1	60.0 ± 7.7	64.5 ± 7.0	70.0 ± 6.5
Male sex (n, %)	817 (76)	194 (72)	214 (80)	206(77)	203 (76)
Duration of diabetes (years)	4.0 (1.0–10.0)	4.0 (1.0–9.0)	4.0 (1.0–9.0)	4.0 (1.0–12.0)	6.0 (1.0–12.0)
HbA1c (%)	6.9 (± 1.1)	6.7 (± 1.0)	7.0 (± 1.3)	6.9 (± 1.1)	7.0 (± 1.1)
HbA1c converted (mmol/mol)	52	50	53	52	53
Fasting blood glucose (mmol/L)	7.9 (6.7–9.5)	7.6 (6.5–8.9)	8.2 (7.0–10.0)	7.7 (6.8–9.3)	7.9 (6.7–9.7)
Total cholesterol (mmol/L)	4.6 ± 1.2	4.2 ± 1.0	4.5 ± 1.1	4.6 ± 1.2	5.0 ± 1.3
HDL-cholesterol (mmol/L)	1.1 ± 0.3	1.2 ± 0.3	1.1 ± 0.3	1.1 ± 0.3	1.1 ± 0.3
LDL-cholesterol (mmol/L)	2.6 ± 1.0	2.2 ± 0.8	2.5 ± 1.0	2.7 ± 1.0	2.9 ± 1.2
Triglycerides (mmol/L)	1.9 ± 1.2	1.7 ± 1.1	1.9 ± 1.1	2.0 ± 1.2	2.1 ± 1.2
non-HDL-cholesterol (mmol/L)	3.31 (2.67–4.17)	2.90 (2.34–3.54)	3.27 (2.66–4.06)	3.48 (2.87–4.31)	3.67 (2.93–4.81)
eGFR (MDRD)	76 ± 21	85 ± 14	83 ± 18	74 ± 21	61 ± 20
Systolic blood pressure (mmHg)	145 ± 21	137 ± 18	144 ± 19	147 ± 22	152 ± 21
Diastolic blood pressure (mmHg)	81 ± 11	81 ± 10	82 ± 11	82 ± 12	79 ± 11
Weight (kg)	85.6 ± 14.9	85.8 ± 15.5	87.2 ± 15.6	86.6 ± 14.3	83.0 ± 13.8
BMI (kg/m^2^)	28.4 ± 4.1	28.3 ± 4.1	28.8 ± 4.6	28.6 ± 4.0	27.7 ± 3.7
Current smoking (n, %)	268 (25)	42 (17)	65 (24)	86 (32)	75 (28)
Microalbuminuria (n, %)	250 (23)	39 (15)	53 (20)	71 (26)	87 (33)
hs-CRP (mg/L)	2.1 (1.0–4.4)	1.1 (0.6–2.3)	2.1 (1.0–4.3)	2.4 (1.4–4.8)	3.4 (1.8–5.7)
Lipid-lowering treatment (n, %)					
Statins	784 (73)	209 (78)	164 (61)	155 (58)	144 (54)
Glucose-lowering treatment (n, %)					
Only lifestyle/diet treatment	233 (22)	49 (18)	71 (27)	57 (21)	56 (21)
Oral treatment	690 (64)	180 (67)	167 (62)	171 (64)	172 (64)
Insulin use	266 (25)	66 (25)	58 (22)	31 (11)	30 (11)
Combination of oral treatment and insulin	116 (11)	27 (10)	28 (10)	24 (9)	27 (10)
Blood pressure medication (n, %)					
β-blockers	616 (57)	184 (69)	149 (56)	145 (54)	138 (52)
Diuretics	371 (35)	65 (24)	82 (31)	104 (39)	120 (45)
ACE inhibitors	444 (41)	112 (46)	103 (38)	108 (40)	111 (41)
Calcium antagonists	277 (26)	58 (22)	73 (27)	67 (25)	79 (30)
Angiotensin II receptor blockers	175 (16)	35 (13)	34 (13)	57 (21)	49 (18)
Antithrombotic therapy (n, %)					
Thrombocyte aggregation inhibitor	833 (78)	223 (83)	208 (78)	202 (75)	200 (75)
Oral anticoagulants	153 (14)	26 (10)	28 (10)	36 (13)	63 (24)
Years since first vascular event	1 (0–9)	0 (0–1)	0 (0–6)	1 (0–10)	9 (1–18)
Location of vascular disease (n, %)					
Cerebrovascular disease	312 (29)	40(15)	59 (22)	75 (28)	138 (49)
Coronary artery disease	734 (68)	215 (80)	188 (70)	167 (62)	164 (61)
Peripheral artery disease	219 (20)	18 (7)	47 (18)	62 (23)	92 (34)
Abdominal aortic aneurysm	73 (7)	1 (0.4)	4 (1.5)	20 (7.4)	48 (18)

Mean age increased from 55.8 ± 7.1 years in the lowest risk quartile to 70.0 ± 6.5 years in the highest risk quartile, and the percentage of males increased from 72 to 76 %. In addition, the mean duration of diabetes increased from 4.0 to 6.0 years and time since first vascular event increased from 0 to 9 years.

### The relation between HbA1c and risk for new cardiovascular events and mortality in the whole cohort

During a mean follow-up period of 6.9 years 243 patients’ deaths were reported. The mean follow-up period for vascular events was 6.4 years, in which period 223 cases were reported. The separate outcomes had follow-up periods of 5.8 years for MI and 6.7 years for stroke, in which period 84 and 48 events were reported, respectively.

In the whole cohort of patients with type 2 diabetes and vascular disease, a 1 % increase in HbA1c level was associated with a higher risk for all-cause mortality (HR 1.18, 95 % CI 1.06–1.31). There was no relation between HbA1c and risk for new cardiovascular events during follow-up (HR 1.03, 95 % CI 0.91–1.16) (Table 2).

**Table 2:** The relation between HbA1c and risk for new cardiovascular events or mortality in the whole cohort and in quartiles stratified by vascular risk

	Whole cohort	Q1	Q2	Q3	Q4
	n = 1096	n = 274	n = 274	n = 274	n = 274
SMART risk score (% 10 year risk for recurrent vascular events)		5–16	16–24	24–37	37–100
All-cause mortality	243 events	21 events	39 events	66 events	117 events
Model I	1.13 (1.02–1.24)	1.42 (0.99–2.04)	1.11 (0.90–1.37)	1.00 (0.82–1.22)	1.23 (1.04–1.46)
Model II	1.21v (1.09–1.34)	1.57 (1.10–2.25)	1.09 (0.87–1.36)	1.04 (0.85–1.27)	1.28 (1.08–1.53)
Model III	1.18 (1.06–1.31)	1.36 (0.93–1.98)	1.04 (0.82–1.33)	1.00 (0.80–1.24)	1.33 (1.11–1.60)
Composite vascular outcome	223 events	28 events	46 events	53 events	96 events
Model I	1.03 (0.92–1.15)	1.23 (0.89–1.70)	0.89 (0.70–1.14)	0.90 (0.71–1.13)	1.18 (0.98–1.42)
Model II	1.07 (0.96–1.20)	1.31 (0.94–1.83)	0.93 (0.73–1.20)	0.94 (0.74–1.19)	1.19 (0.99–1.43)
Model III	1.03 (0.91–1.16)	1.17 (0.83–1.67)	0.92 (0.71–1.20)	0.88 (0.68–1.15)	1.20 (0.99–1.46)
Myocardial infarction	84 events	15 events	23 events	23 events	23 events
Model I	0.88 (0.72–1.08)	0.86 (0.50–1.49)	0.70 (0.47–1.06)	0.94 (0.70–1.32)	1.02 (0.68–1.53)
Model II	0.88 (0.72–1.09)	0.87 (0.50–1.52)	0.71 (0.47–1.08)	0.99 (0.70–1.40)	0.99 (0.66–1.49)
Model III	0.87 (0.71–1.07)	0.92 (0.51–1.65)	0.71 (0.46–1.10)	1.01 (0.69–1.48)	0.99 (0.66–1.47)
Ischemic stroke	48 events	5 events	10 events	9 events	24 events
Model I	1.10 (0.88–1.39)	1.57 (0.81–3.01)	1.09 (0.70–1.70)	0.66 (0.34–1.29)	1.25 (0.88–1.79)
Model II	1.16 (0.92–1.48)	1.72 (0.87–3.40)	1.22 (0.77–1.95)	0.64 (0.33–1.25)	1.27 (0.89–1.83)
Model III	1.09 (0.84–1.41)	2.13 (0.79–5.75)	1.28 (0.77–2.13)	0.60 (0.31–1.19)	1.26 (0.86–1.85)
Cardiovascular mortality	140 events	11 events	22 events	30 events	77 events
Model I	1.07 (0.94–1.23)	1.41 (0.85–2.35)	0.91 (0.64–1.29)	0.97 (0.72–1.30)	1.21 (0.98–1.49)
Model II	1.15 (1.00–1.32)	1.62 (1.00–2.62)	0.92 (0.64–1.31)	1.00 (0.74–1.34)	1.24 (1.00–1.54)
Model III	1.09 (0.94–1.27)	0.97 (0.59–1.60)	0.79 (0.52–1.21)	0.94 (0.67–1.31)	1.29 (1.03–1.61)

The relation between HbA1c and risk for each outcome under study is represented separately. Model I is the crude model, Model II is adjusted for sex and age, and Model III is additionally adjusted for current smoking, non-HDL cholesterol, diabetes duration, systolic blood pressure and eGFR (MDRD). The hazard ratios are given per 1 % HbA1c. For example, in the patients of this cohort a 1 % higher HbA1c is associated with a 1.18-fold higher risk of all-cause mortality

### The relation between HbA1c and new cardiovascular events and mortality according to baseline risk

**Fig. 1:**
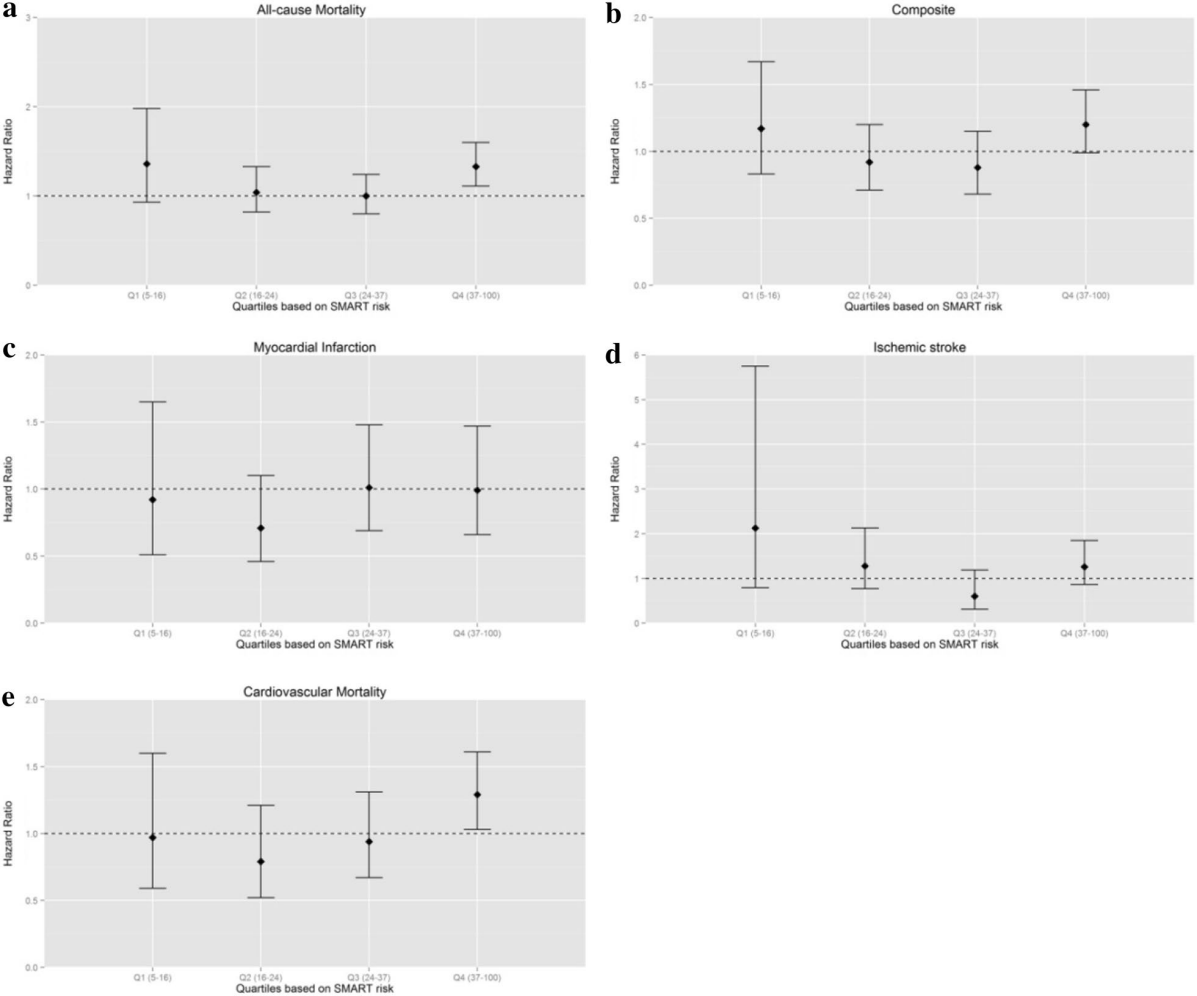
The relation between HbA1c and risk for new cardiovascular events or mortality in quartiles stratified by baseline risk. The relation between HbA1c and risk for each outcome under study is represented separately. The quartiles are denoted on the x axis with Q1–Q4 respectively. The SMART risk score range of each quartile is given between *brackets*. Hazard ratios are adjusted for sex, age, current smoking, non-HDL cholesterol, diabetes duration, systolic blood pressure and eGFR (MDRD). Figure 1 **a**–**e** show the association between the SMART risk score and risk for a specified cardiovascular event (**b**, **c**, **d**) or mortality (**a**, **e**). The SMART risk score calculates the 10-year risk for developing a new cardiovascular event in patients with a history of cardiovascular disease. For example, a person with a SMART risk score of 20 has a 20 % chance of experiencing a new cardiovascular event in the upcoming 10 years

In patients with the highest predicted 10 years risk for new cardiovascular disease or mortality (37–100 %), a 1 % increase in HbA1c was associated with an increased risk for cardiovascular mortality (HR 1.29, 95 % CI 1.03–1.61) and all-cause mortality (HR 1.33, 95 % CI 1.11–1.60) (Table 2). The results for the lowest SMART risk quartile were similar, albeit not significant (HR 1.36, 95 % CI 0.93–1.98). An inverse relation was suggested between HbA1c and risk for MI in all quartiles (Fig. 1). This was most apparent in the second risk quartile (HR 0.71, 95 % CI 0.46–1.10).

However, the interaction term between HbA1c and the SMART risk score was not significant for the composite cardiovascular outcomes and for cardiovascular or all-cause mortality (*p* = 0.225, *p* = 0.259 and *p* = 0.179 respectively). Thus, baseline risk as estimated by the SMART risk score did not modify the relation between HbA1c and cardiovascular events or mortality in patients with type 2 diabetes and cardiovascular disease.

None of our sensitivity analyses changed the direction or magnitude of the modifying effect of the SMART risk score on the relation between HbA1c and vascular outcomes and mortality.

## Discussion

In the present study we show that within a population of patients with type 2 diabetes and CVD at baseline, an increase in HbA1c is related to an increased risk for all-cause mortality, but not to an increased risk for cardiovascular events. The relation between HbA1c and risk for all-cause mortality is not significantly influenced by baseline vascular risk.

The Veterans Affairs Diabetes Trial (VADT) study also stratified their patients with type 2 diabetes, of which 38 % had CVD at baseline, by baseline risk, using coronary artery calcium (CAC) as a measure for atherosclerosis [25]. Strict glycaemic control was related to lower incidence of future cardiovascular events in patients with a CAC score below 100 at baseline, while this effect was not present in patients with higher CAC values [25]. Although not directly comparable to our study due to the interventional nature of the VADT, these findings do suggest that within a high risk population, the relation between glycaemic control and cardiovascular events differs according to risk for (new) cardiovascular events.

The absence of a relation between HbA1c and (new) vascular disease in our study population could be related to differences in the pathogenesis of vascular disease in patients with type 2 diabetes. In the later stages of type 2 diabetes other risk factors than glycaemic control may be more important in inducing vascular complications. These include hypertension [26, 27], lipids [28, 29], and the calcification of the intima media of blood vessels, called Mönckeberg’s sclerosis [30]. The latter condition is strongly associated with morbidity and mortality in diabetes and is independent of glycaemic control [30].

Following this line of reasoning, the significant relation between HbA1c and mortality in the whole cohort is an unexpected finding. This result may be powered by the patients in the highest risk quartile, who are older and have a longer history of vascular disease and diabetes. In this group of high risk patients, a high HbA1c may be a proxy of frailty. Indeed, higher HbA1c levels are related to a higher chance of being frail in women, and this relation is strongest above an HbA1c level of 9 % (75 mmol/mol) [31]. Frailty in patients with diabetes is associated with higher mortality and glucose dysregulation [32], and the prevalence of frailty increases with age.

We propose that in the highest risk quartile, HbA1c might be regarded as a proxy for overall health status or frailty. High HbA1c levels would then reflect a poor overall health status, which in itself can be regarded as a risk factor for mortality. Although the whole study population had a mean HbA1c of 51.9 mmol/mol, there was a significant distribution of HbA1c levels (range 29–116.4 mmol/mol) in the whole cohort. While it is quite possible that the relation between HbA1c and endpoints is different in very high HbA1c levels (for example >100 mmol/mol), our study lacks the statistical power to address this question. In that regard, our study population is an example of a cohort of patients with type 2 diabetes and cardiovascular disease which is comparable to patient population typically seen in an everyday out-patient clinic.

The main strengths of this study include the prospective design, the substantial follow-up period and large cohort size providing a relatively high number of events. Furthermore, the completeness of data reduced the risk of bias. The use of the SMART risk score, which was derived from the SMART cohort, ensured that the risk score used was well suited for the dataset. However, it should be noted that this score is not yet externally validated.

Several limitations of this study need to be addressed. The size of the study population limited the amount of analyses we could perform. As a result of the stratification we performed, some outcomes lost analytic power. This was specially the case for the lower risk quartiles, in which few events occurred during the study period. Validation of our findings in larger cohorts would aid to support our conclusions. As the current study was conducted in an observational cohort, no conclusions regarding treatment effects could be made. In addition, limited information about microvascular outcomes was available and these could therefore not be addressed in the current study. Our suggestion of a link with frailty could not be supported by our data due to the absence of data on frailty status. As precise information on non-cardiovascular causes of death was lacking, we could not evaluate whether the relation between HbA1c and all-cause mortality might still be powered by presumed diabetes-related non-cardiovascular cause of death like cirrhosis associated with non-alcoholic fatty liver disease.

## Conclusions

In conclusion, in patients with type 2 diabetes and CVD, HbA1c is related to the risk of all-cause mortality, but not to the risk of cardiovascular events. The relation between HbA1c and all-cause mortality in patients with type 2 diabetes and vascular disease is not dependent on baseline vascular risk.

## Abbreviations

CAC: coronary artery calcium
CVD: cardiovascular disease
hs-CRP: high-sensitivity C-reactive protein
MI: myocardial infarction
SMART: second manifestations of ARTerial disease
VADT: veterans affairs diabetes trial
